# Targeting Growth Factor Signaling Pathways in Pancreatic Cancer: Towards Inhibiting Chemoresistance

**DOI:** 10.3389/fonc.2021.683788

**Published:** 2021-06-14

**Authors:** Ntombikayise Xelwa, Geoffrey Patrick Candy, John Devar, Jones Omoshoro-Jones, Martin Smith, Ekene Emmanuel Nweke

**Affiliations:** Department of Surgery, University of Witwatersrand, Johannesburg, South Africa

**Keywords:** pancreatic cancer, chemoresistance, growth factors, chemotherapy, signaling pathways

## Abstract

Pancreatic cancer is one of the most deadly cancers, ranking amongst the top leading cause of cancer related deaths in developed countries. Features such as dense stroma microenvironment, abnormal signaling pathways, and genetic heterogeneity of the tumors contribute to its chemoresistant characteristics. Amongst these features, growth factors have been observed to play crucial roles in cancer cell survival, progression, and chemoresistance. Here we review the role of the individual growth factors in pancreatic cancer chemoresistance. Importantly, the interplay between the tumor microenvironment and chemoresistance is explored in the context of pivotal role played by growth factors. We further describe current and future potential therapeutic targeting of these factors.

## Introduction

In developed countries, pancreatic cancer (PC) is poised to become amongst the top three causes of deaths in cancer patients by 2030 (1, 2). PC is characterized by non-specific symptoms especially at the early stages of the cancer enabling the progression of the disease. Due to late diagnosis and the biology of PC, current drug treatments usually lead to poor tumor response rates and early relapse (3, 4). Pancreatic cancer cells produce growth factors that play various key roles in propagating tumorigenesis, one of which is in chemoresistance (5). In PC treatment and management, chemotherapy remains a well-known mode of treatment. The lack of effective chemotherapeutic agents to treat PC contribute significantly in making it a lethal condition.

## Chemoresistance in PDAC: Role of the Tumor Microenvironment

Many features contribute to chemoresistance and tumor progression, with the presence of a pro-tumoral microenvironment being the dominant factor (6, 7). It has been observed that 90% of the total tumor volume comprises the desmoplastic stroma arising due to the pro-fibrotic state initiated by the PC cells and microenvironment. This composition ensures that PC cells are chemoresistant by reducing drug delivery into the tumor microenvironment (8). Due to this, PC cells can develop resistance towards standard of care chemotherapeutic drugs such as gemcitabine used for PC treatment (9). Gemcitabine is a deoxycytidine analog, that functions by intercalating with the DNA, consequently blocks the cell cycle at the G1/S phase, and inhibits cellular proliferation. However, this blockage may be impaired by changes in drug transporters, activation and inactivation of enzymes and their targets that are characteristic of chemoresistance (10).

The tumor microenvironment of PC which consists of cells such as tumor-associated macrophages (TAMs), myofibroblasts, stromal cells, and cancer-associated fibroblasts (CAFs), is a key player in inducing chemoresistance (11–19). For example, CAFs have been implicated in drug resistance in pancreatic cancer *via* several mechanisms such as upregulating cytokine levels, blocking adequate delivery of drugs, activating tumor-promoting signaling pathways, increasing cell proliferation, promoting metastasis, and dysregulating cellular metabolism. Recently, novel strategies to target them are being developed (20–24). Most proteins within the intricate extracellular matrix (ECM) of PC are secreted by CAFs (20, 25); thus, the dominance and heterogeneity of CAF within the TME guarantees that it plays a pivotal role in therapeutic resistance (20, 26). Drug delivery to tumor cells is essential for drug sensitivity and therapeutic efficacy. In PC, the dense TME architecture ensures that drug delivery is impeded. Our group recently showed using SWATH-MS, the intricate network of signaling pathways within the ECM of PC tumors highlighting different known and potentially novel associating proteins (27). Furthermore, mouse models of PC showed that CAFs accumulate gemcitabine and this may contribute to drug resistance (28). Also, CAFs originate from pancreatic stellate cells (PSCs) and these cells can secrete metabolites such as deoxycytidine which confers resistance on PC cells (29).

TAMs can either function as inflammatory macrophages known as M1-like inflammatory macrophages which are tumor inhibiting or as the tumor-promoting M2-like immunosuppressive macrophages, both of which play important roles in solid tumors such as pancreatic cancer (30–32). The M1 and M2-like macrophages can be described as either tumor killing or promoting, respectively. Chemoresistance in PC has also been linked to pancreatic cancer stem cells which have enhanced epithelial-mesenchymal transition (EMT), altered metabolism, altered key genes (such as *KRAS*, *TP53*, *CCND1*, *BIRC5*, and *BCL-2*), dysregulated signaling pathways (including Notch, PI3K/AKT, Hedgehog, and NF-κB), reduced apoptosis, and increased cell cycle (33, 34).

## Growth Factors and Chemoresistance

Growth factors are associated in the progression of several cancers including pancreatic cancer, leading to the development of several drugs to target them (5, 35). The functions of the various growth factors in promoting chemoresistance in pancreatic cancer, current drugs targeting them, and clinical trials are further elaborated on.

### Insulin-Like Growth Factor (IGF)

The IGF pathway is dysregulated in pancreatic cancer and its secretion has been linked to the tumor microenvironment (36, 37). Recent studies have shown its dysregulation in both tumors and blood samples of pancreatic cancer patients (38, 39). Furthermore, the upregulation of the insulin/IGF-1R pathway in PDAC occurs in about 72% of patients and is associated with an increase in the number of CD163^+^TAMs (12). The Ireland et al. (2016) study reported on the role of TAMs and myofibroblasts in promoting drug resistance in pancreatic cancer by the secretion of IGF 1 and 2. The secretion of these factors desensitized pancreatic cancer cells to gemcitabine as they showed that by blocking the IGF pathway, gemcitabine became effective. Also, fibroblast exposed to pancreatic cancer cells has also been shown to secrete the IGF contributing to survival and proliferation (36). When IGF-1R was silenced in PC cells, cell growth, proliferation, and metastasis were inhibited (40). Supporting evidence revealed that blocking IGF-1R and ErbB3 sensitized tumor cells to nab-Paclitaxel and gemcitabine (41). The efficacy of another IGF-1R inhibitor, ganitumab, was evaluated in a phase II clinical trial and found to improve the overall survival of metastatic PC patients over a period of 6 months while ensuring manageable toxicity levels (42). In another study, although combination of ganitumab and gemcitabine resulted in admissible toxicity levels, it did not improve overall survival (43). In *in vivo* models, the simultaneous inhibition of IGF-1R and ErbB3 using istiratumab (MM-141) blocked the PI3K/AKT/mTOR axis thus increasing the efficacy of blocking IGF-1R alone or in combination with other agents such as gemcitabine (44). On the other hand, a combinatorial treatment of istiratumab with gemcitabine and nab-paclitaxel did not improve outcomes in metastatic pancreatic cancer patients compared to standard of care chemotherapy (45). However there are currently 136 ongoing clinical trial investigating the efficacy of nab-paclitaxel alone or in combination with other drugs such as FOLFIRINOX (**Table 1**).

**Table 1:** Ongoing clinical trials evaluating growth factor inhibition in pancreatic cancer.

Drugs/Inhibitors	Growth factor target	Number of ongoing clinical trials with results*	Status of clinical trials	Clinical trial number
Nab-Paclitaxel	IGF-1R	142	41 active, not recruiting 101 recruiting	Such as NCT03316599, NCT03520790, NCT02827201, NCT02210559, NCT02047513, NCT03086369, NCT02717091, NCT02481635, NCT03252808, NCT02427841, NCT04808687, NCT02340117, NCT03929094, NCT03850769, NCT03941093, NCT03885219, NCT03652428, NCT03636308, NCT04498689,
Bevacizumab	VEGF	13	7 active, not recruiting6 recruiting	NCT03387098, NCT03329248, NCT03136406, NCT03586869, NCT03376659, NCT01229943, NCT04299880 NCT03351296, NCT03597581, NCT03193190, NCT02820857, NCT04430842, NCT03597581
Sunitinib	VEGFR, PDGFR,	4	4 recruiting	NCT02230176, NCT02282059, NCT02465060, NCT03878524,
Nintedanib	VEGFR, FGFR, PDGFR	1	1 recruiting	NCT02902484
Erlotinib	EGFR	6	4 active, not recruiting 2 recruiting	NCT01013649, NCT00878163, NCT01660971, NCT02737228 NCT04136132, NCT03878524
Cetuximab	EGFR	4	3 active, not recruiting 1 recruiting	NCT03992664, NCT03319459, NCT01420874 NCT03785249
Imatinib	PDGFR	1	1 recruiting	NCT03878524
Pamrevlumab (FG-3019)	CTGF	2	1 active, not recruiting 1 recruiting	NCT02210559 NCT03941093
Larotrectinib	NGF	1	1 recruiting	NCT02465060
Ficlatuzumab	HGF/c-MET	1	1 active, not recruiting	NCT03316599
Crizotinib	HGF/c-MET	2	2 recruiting	NCT02465060, NCT02568267

*Studies could involve treatment alone or in combination with other therapeutic strategies.

### Vascular Endothelial Growth Factor (VEGF)

**Figure 1:**
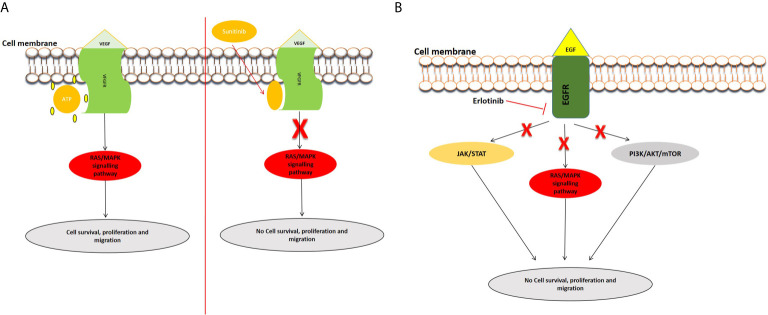
Examples of inhibition of the VEGFR and EGFR signaling pathways. **(A)** Inhibition of VEGFR signal transduction by sunitinib. After the entry of sunitinib into the cytoplasm, it competitively binds at the ATP site of VEGFR, consequently inhibiting the activation of the pathway. **(B)** Mechanism of action of Tyrosine kinase inhibitor, erlotinib. Erlotinib is a small molecule that acts as an ATP analogue and inhibits EGF signaling by binding to receptor tyrosine kinases (RTKs), and inhibits the activation of downstream signaling pathways.

Angiogenesis is required for solid tumor development and progression (46). The heparin-binding glycoprotein, VEGF, functions as an endothelial cell mitogen and is strongly linked to angiogenesis in different tumors, including pancreatic cancer (47). VEGF is highly expressed in PC and many studies have determined that its overexpression, correlated to greater tumor size, increased liver metastases, and a reduced patient survival (48–54). Several preclinical and clinical studies have evaluated the efficacy of inhibiting VEGF and its receptors in PC (54–57). For example, the inhibition of VEGF/VEGFR by foretinib blocked angiogenesis and cell proliferation and resulted in increased apoptosis (58). Bevacizumab (an anti-VEGF antibody) and Sunitinib [an anti-tumor and anti-angiogenic tyrosine kinase (TKI) inhibitor] were observed to inhibit PC cell motility and migration (54). Sunitinib blocks VEGFR, allowing angiogenesis to be inhibited in pancreatic cancer cells (59) (**Figure 1A**). Treatment with cedirinib, a VEGF inhibitor, was also found to reduce the expression of key epithelial-to-mesenchymal transition (EMT) markers such as ZEB1, Snail, and N-cadherin (60), suggesting potential roles in inhibiting cancer cell migration and metastasis. The combination of VEGF inhibitors such as bevacizumab and chemotherapeutic agents like gemcitabine and 5-FU have shown promising potential in treating PC (61).

### Epidermal Growth Factor (EGF)

Epidermal growth factor receptor (EGFR) is expressed in up to 60–90% of pancreatic cancers and is involved inducing cell growth and migration. Targeted anti-EGFR molecular strategies have been employed in the treatment of PDAC especially to circumvent chemoresistance (62, 63). Erlotinib, one of the most studied EGFR inhibitors, has been used to block the EGFR signaling pathway (**Figure 1B**). A phase I and II clinical trial concluded that erlotinib might be an effective and safe treatment option in PC (64). Combinational treatment of erlotinib with gemcitabine prolonged survival in pancreatic cancer patients in a phase III study (65). Simultaneous treatment of erlotinib, gemcitabine, and capecitabine also showed efficacy in metastatic PC patients (66). Additionally, studies have demonstrated the use of statins in combination with anti-EGFR agents in pancreatic cancer treatment (67). Statins, known to lower lipid concentrations, block the production of intermediates needed for prenylation and RAS/mitogen-activated protein kinase 1 signaling activation. They appear to alter resistance to anti-EGFR agents, such as erlotinib and show efficacy when combined with drugs like gemcitabine (68, 69).

### Fibroblast Growth Factor (FGF)

The fibroblast growth factors (FGFs) include about 23 known proteins and their receptors are associated with PC playing a role in tissue hyperplasia, transition of EMT, tumor metastasis, and angiogenesis (70). FGF is overexpressed in PC and promotes cell growth, proliferation, and invasion (71, 72). The overexpression of FGF and FGFR can lead to oxidative stress evident through increased nitric oxide synthase (iNOS) (73). FGF10 is a molecule involved in mesenchymal-epithelial signaling and is crucial in development of multiple organs including the pancreas (74). However, when alterations occur, the FGF10 can induce migration and invasion in the PC cells. As the mesenchyme is essential for the pancreas growth, its absence can result in lack of islet cells and hypoplasia. Furthermore, the crosstalk between the FGF10 and TGF-β pathway can promote EMT and cancer cell invasion (75, 76). A recent study observed that a high expression of FGF8 was independently associated with diminished overall patient survival, indicative of poor prognosis (77). The FGF can also play a role in chemoresistance. It was determined that, acquired drug resistance observed when tumors were treated with VEGF inhibitors was induced by several factors including hypoxia and FGF expression (78). FGF2 targeting agents and inhibitors can aid in preventing TAM-associated cell migration and chemoresistance (11, 73). One study evaluated the use of dovitinib, an inhibitor of FGFR/VEGFR pathway in combination with gemcitabine and capacitabine and determined improved efficacy in patients with advanced PC (79). Mastinib, another inhibitor of FGFR and PDGFR, was shown to decrease inflammation in PC patients (80). Combining mastinib and gemcitabine in both *in vitro* and *in vivo* PC models showed that mastinib sensitized tumor cells to gemcitabine (81). This was further supported by a phase III clinical trial which indicated the use of the combination of mastinib and gemcitabine in patients that overexpress *ACOX1* (82).

### Transforming Growth Factor-Beta (TGF‐β)

Mutations of key genes such as *SMAD4* are a major characteristic of PC initiation and progression. These mutations can be found in more than half of pancreatic cancer patients and play an important role in TGF-β signaling (83, 84). In the TME, TGF‐β signaling pathway is involved in the regulation of several cell types. For instance, it promotes differentiation of myofibroblasts, recruitment of immune cells and influences epithelial and endothelial cell differentiation (85). This pathway also has contradictory functions as a tumor suppressor and promoter (86). During early tumorigenesis, TGF-β signaling can inhibit cell cycle and induce apoptosis, but could also promote tumor growth *via* enhancing EMT, cancer stem cells formation, cellular migration, invasion, and immune response evasion by inhibiting Th1 immune response (87). Cancer cells can secrete both PDGF-BB and TGF-β leading to promotion of fibroblasts transformation thereby resulting in the low expression of the anti-cancer Pigment Epithelium-Derived Factor (PEDF) (88).

Another study determined that silencing TGF-βRII, the protein that begins the TGF-β signaling, promoted tumor growth and resistance to gemcitabine (89). On the other hand, several inhibitors of the TGF-β signaling pathway such as trabedersen and galunisertib have been developed and tested in pancreatic cancer with the aim of inhibiting tumor growth (90, 91). LY2109761, an inhibitor of TGF-β receptors I and II, was used alone and in combination with gemcitabine and revealed to inhibit cell survival, migration, and metastases (92). Cisplatin (Platin) resistance can also be promoted through altering TGF-β/SMAD4 signaling and up-regulation of EMT-markers by tumor-derived exosomes (TDEs), known to function in development and progression of several biological processes in cancer (93). It was shown that the release of TGF‐β and FGF5 from CAFs causes myofibroblast reprogramming in cancer stem cells (CSCs), which is needed to protect the cells from external influences and to acquire a chemoresistance characteristic within the cells (94).

### Connective Tissue Growth Factor

Connective Tissue Growth Factor (CTGF/CCN2) is a protein found in the extracellular matrix that functions in regulating diverse cellular processes such as cell survival, proliferation, migration, and apoptosis (95). CTGF has also been linked to other growth factor signaling pathways including TGF-β and FGF. Studies showed that TGF-β can prompt CTGF production in PC cell lines and it can induce the expression of FGFR2 (96, 97). Over recent years, CTGF has been increasingly investigated as a target for PC therapies (98). *In vivo* silencing of CTGF resulted in the reduction of tumor growth and its expression was closely associated with hypoxia and density of tumor-surrounding stromal cells (99). Treatment with an antagonist of CTGF, mAbFG-3019, using murine models of pancreatic ductal adenocarcinoma revealed that gemcitabine-based chemotherapy was enhanced by increasing levels of gemcitabine and leading to reduction of tumor size (100). The efficacy of pamrevlumab (FG-3019) against pancreatic tumors was further confirmed by another study that showed its effectiveness in selectively targeting pancreatic tumor cells and inhibiting metastases (101). In a phase I and II study, pamrevlumab, enhanced gemcitabine activity in locally advanced pancreatic cancer patients (102). Similarly, another study also demonstrated the use of peptides targeting CTGF alone and in combination with gemcitabine in reducing tumor size (103).

### Platelet-Derived Growth Factor (PDGF)

The PDGF family can bind to the tyrosine kinase receptors, PDGFRα and PDGFRβ, and interacts with different cell types within the tumor microenvironment to enhance tumor progression and chemoresistance (104, 105). PDGF regulates PC progression by mediating pathways such as HIPPO/Yes and *via* its interaction with DUSP28 (106, 107). Inhibiting the phosphorylation of PDGFR by using Gleevec (Imatinib) with gemcitabine led to a reduction in pancreatic tumor growth in nude mice models (108). The tyrosine kinase inhibitor (Sunitinib) targets both VEGFR1-3 and PDGFR pathways (109). Sunitinib appears to be a promising drug in instances where patients did not respond well to gemcitabine-based treatments (110). In another study, the use of a multi-kinase inhibitor, nintedanib, which simultaneously targets VEGFR, FGFR, and PDGFR signaling was investigated alone, and in combination with gemcitabine in xenograft models and this drug displayed a strong antitumor activity (111).

### Nerve Growth Factor (NGF)

Nerve growth factor (NGF) is present in sympathetic and neural crest-derived sensory neurons, as well as in the central nervous system (CNS) (112). Pancreatic cancer cell can invade surrounding nerve cell spaces leading to perineural invasion. Perineural invasion is common in pancreatic cancer cells correlating to poor prognosis and can be facilitated by nerve growth factor (113–115). Overexpression of NGF and BDNF induces noradrenaline accumulation consequently inducing pancreatic cancer cell growth (116). Due to its role in inhibiting apoptosis, NGF has also been implicated in chemoresistance (117). Time-dependent treatment of PC mouse models determined that depleting NGF inhibits inflammation and metastasis (118). The inhibition of NGF by blocking STAT3 resulted in decreased pancreatic cancer migration and reduced perineural invasion (119). Similarly, another study showed that NGF knockdown prevented pancreatic cancer cell proliferation, invasion, and migration (120).

### Hepatocyte Growth Factor (HGF)

Together with its receptor, c-MET, HGF is overexpressed in PC and has been linked to cancer cell invasion, metastasis, and chemoresistance *via* tumor-promoting pathways such as P13/Akt and neuropilin (121–125). HGF levels predicted overall survival in locally advanced PC patients that underwent neoadjuvant therapy (126). One study demonstrated the promotion of PC cells by pancreatic stellate cells through the HGF/c-MET pathway; this pathway required survivin expression and was regulated by the p53/p21 pathway (127). Several inhibitors such as rilotumumab, ficlatuzumab onartuzumab, crizotinib, tivantinib, foretinib, and cabozantinib can be used against HGF/c-MET (128). For example, blocking HGF using rilotumumab *in vivo* led to decreased metastasis compared to treatment with gemcitabine (129). Patients treated with the combinatorial therapy of ficlatuzumab nab-paclitaxel and gemcitabine had favorable treatment response albeit with an observed significant decrease in albumin levels and increased body swelling (130). A synergistic effect was observed when PC cell cultures were treated with tivantinib and gemcitabine, suggesting their possible use in patient treatment (131).

## Conclusion and Future Prospects

Chemoresistance is rampant in pancreatic cancer. Growth factors play a crucial role in chemoresistance. Efficient elucidation of growth factor signaling and associated pathways in initiating and propagating chemoresistance is crucial in targeting these pathways. In this instance, the investigation of the interplay of growth factor signaling pathways and other upstream/downstream pathways might provide us with better understanding of chemoresistance in pancreatic cancer and how to circumvent it. Of importance is the elucidation of the diverse roles these growth factors play in the tumor microenvironment. Targeting these growth factors by the use of pharmaceutical agents and inhibitors individually may prove difficult. Perhaps the use of combination therapy targeting the various growth factors may be more effective. For example, blocking FGFR/PDGFR/VEGFR increased survival in mouse models, slowed tumor growth and increased effectiveness of gemcitabine on pancreatic cancer cells (35). Additionally, combining growth factor inhibitors with chemotherapeutic drugs such as gemcitabine may facilitate synergistic drug action and improve chemosensitivity. Although, several preclinical and clinical trials are needed as it is possible that some drug combination may be antagonistic as shown in one study where HGF inhibitor and gemcitabine was combined (132). Toxicity levels are also of concern with combinatorial treatments, while efficacy towards tumor treatment might be achieved, the tolerable toxicity levels might be exceeded. Another promising strategy would be the combination of growth factor inhibitors with peptide inhibitors, statins, or non-coding RNAs inhibitors. This type of strategy was demonstrated when the simultaneous silencing of miR-21 and sunitnib treatment resulted in a synergistic increase in anti-tumor effects (133). In conclusion, growth factor inhibition is promising in pancreatic cancer treatment and management which is evident from the many studies discussed here and the various ongoing clinical trials evaluating their efficacies.

## Author Contributions

NX, GC, MS, and EN drafted and conceptualized the article. NX, GC, JD, JO-J, and MS critically edited the article. All authors contributed to the article and approved the submitted version.

## Funding

These laboratories of the authors are supported by the National Research Foundation (NRF), the Cancer Association of South Africa (CANSA), and the South African Medical Research Council grant awarded to the Wits Epithelial Cancer thrust.

## Conflict of Interest

The authors declare that the research was conducted in the absence of any commercial or financial relationships that could be construed as a potential conflict of interest.
